# Design and Optimization of PEDOT/Graphene Oxide and PEDOT/Reduced Graphene Oxide Electrodes to Improve the Performance of Microbial Fuel Cells, Accompanied by Comprehensive Electrochemical Analysis

**DOI:** 10.3390/polym16223134

**Published:** 2024-11-10

**Authors:** Gean Arteaga-Arroyo, Andrea Ramos-Hernández, Aldeir De Los Reyes-Rios, Maximiliano Méndez-López, Karina Pastor-Sierra, Daniel Insuasty, Edgar Marquez, Jayson Fals

**Affiliations:** 1 Grupo de Investigación Biomédica y Biología Molecular, Facultad de Ciencias de la Salud, Universidad del Sinú, Montería 230001, Colombia; geanarteaga@unisinu.edu.co (G.A.-A.); karinapastor@unisinu.edu.co (K.P.-S.); 2 Grupo Química Supramolecular Aplicada, Semillero Electroquímica Aplicada, Facultad de Ciencias Básicas, Programa de Química, Universidad del Atlántico, Barranquilla 081007, Colombia; andrearamos@mail.uniatlantico.edu.co (A.R.-H.); aadlrr@hotmail.com (A.D.L.R.-R.); 3 Grupo de Química y Biología, Departamento de Química y Biología, Universidad del Norte, Barranquilla 081007, Colombia; insuastyd@uninorte.edu.co (D.I.); ebrazon@uninorte.edu.co (E.M.); 4 Grupo de Investigación en Oxi/Hidrotratamiento Catalítico y Nuevos Materiales, Programa de Química, Facultad de Ciencias Básicas, Universidad del Atlántico, Barranquilla 081007, Colombia; jaysonfals@mail.uniatlantico.edu.co

**Keywords:** microbial fuel cells, *Methanococcus deltae*, PEDOT, graphene oxide

## Abstract

A comprehensive investigation into the design and electrochemical optimization of composite electrodes consisting of poly(3,4-ethylenedioxythiophene) (PEDOT)/graphene oxide (GO)/*Methanococcus deltae* and reduced graphene oxide (rGO)/*Methanococcus deltae* hybrids, anchored onto stainless-steel (SS) substrates, has been conducted. The GO and rGO materials were synthesized using a modified Hummer method. The resulting SS/PEDOT/GO and SS/PEDOT/rGO composite electrodes were subjected to systematic electrochemical characterization, focusing on the PEDOT p-type and n-type doping/undoping processes within diverse solvent environments (CH_3_CN and H_2_O) and electrolyte compositions (LiClO_4_ and KCl). Raman spectroscopy analysis confirmed the successful integration of graphene derivatives into the electrode structures, while field-emission scanning electron microscopy (FESEM) and atomic force microscopy (AFM) revealed increased surface roughness upon GO and rGO incorporation. This increase in surface roughness is believed to enhance the adhesion of *Methanococcus deltae* microorganisms and facilitate efficient electron transport. Electrochemical measurements showed that the resulting SS/PEDOT/GO and SS/PEDOT/rGO anodes exhibit remarkable electrocatalytic activity. The SS/PEDOT/GO electrode achieved a maximum power density of 1014.420 mW/cm^2^, while the SS/PEDOT/rGO electrode reached 632.019 mW/cm^2^.

## 1. Introduction

In recent decades, the depletion of non-renewable energy sources has prompted the search for new, environmentally sustainable resources. This search aims to minimize the impact on nature and combat pollution. Microbial fuel cells (MFCs) have emerged as a promising alternative for energy production and are being studied intensively by many researchers [1,2,3,4]. These innovative devices use microorganisms to generate electricity, break down organic matter, and biologically clean up polluted environments. Fundamentally, MFCs work on the principle that microorganisms can either accept or donate electrons to organic and/or inorganic compounds within a given substrate. This electron transfer process takes place at the anode or cathode (electrodes), with direct electron transfer (DET) and mediated electron transfer (MET) being the primary mechanisms studied [5,6].

The electrode used in MFCs must have specific characteristics, such as high conductivity to improve energy production, high surface area and porosity, non-toxicity to microorganisms, stability, durability, and cost-effectiveness [7,8,9]. Carbonaceous materials, particularly stainless steel (SS), have been predominantly used as anode electrodes because they partially meet these criteria [7,10]. However, the use of bare electrodes has not been sufficient to establish MFCs as a reliable and sustainable source of electrical energy. Therefore, electrode modification needs to be explored to make MFCs a consistent, viable alternative with improved energy harvesting capability.

Conductive polymers (CPs) are a class of materials that have attracted considerable interest for their potential application in the modification of electrodes used in MFCs. CPs are known for their exceptional electronic conductivity, comparable to that of metals and semiconductors, and for their versatility, which allows them to be easily integrated into chemical sensors and electronic devices [11,12]. In addition, CPs offer a flexible synthesis process that allows the use of chemical or electrochemical routes. The electrochemical stability of CPs in both reduced and oxidized states is a crucial parameter that determines their lifetime and suitability for technological and biotechnological applications, including MFCs [13,14].

The primary goal for using CPs in microbial fuel cells is to improve the electronic conductivity of anodic materials while increasing the active surface area, without compromising other critical factors essential for optimal device performance [15]. Historically, research efforts have mainly focused on exploring the potential of specific conductive polymers, such as polypyrrole (PPy), polyaniline (PANI), polythiophene (PTh), and poly(3,4-ethylenedioxythiophene) (PEDOT), for integration into MFC systems [7,8].

PEDOT stands out among CPs for its exceptional conductivity due to its low band gap, which allows reversible electrochemical p- and n-doping. Notable for its good biocompatibility and high thermal and electrochemical stability, PEDOT has attracted attention for its promising properties in MFC applications [7,16,17,18]. Studies have reported that the synergy between PEDOT and various anode structural configurations (such as graphite plate, carbon cloth, and graphite felt) significantly enhances electrode performance when MFC anodes are coated with PEDOT [19]. Additionally, an optimal PEDOT loading of 2.5 mg/cm^2^ was identified, yielding a maximum current density of 3.5 A/m^2^ and a coulombic efficiency of 51% [20]. Another study investigated the modification of stainlesssteel (SS) plate electrodes with PEDOT, resulting in increased surface area and porosity, which increased the electrode capacity for biofilm growth and facilitated electron transfer [21]. Numerous studies have demonstrated the efficacy of PEDOT-coated electrodes in microbial devices, showing improved electron transfer processes, increased surface area, improved porosity, and compatibility with microorganisms, contributing to greater efficiency in MFCs [22,23].

Beyond the application of PEDOT to bare electrodes, researchers have ventured into decorating or combining PEDOT with other materials to create novel composites, with the aim of achieving improved performance in MFCs. For example, PEDOT polystyrene sulphonate (PEDOT-PSS)-modified electrodes were successfully synthesized electrochemically, and their performance as anodes in urine-fed MFCs was evaluated. These PEDOT-PSS electrodes showed superior performance compared to bare carbon veil electrodes, significantly improving electron transfer and facilitating biofilm growth [24].

Graphene (G)-based composites have been integrated into MFCs due to their favorable mechanical and environmental properties and low cost, which are well suited to the electrode requirements of MFCs [8,25,26,27,28,29]. Composites combining graphene with CPs have attracted considerable interest due to the synergies they offer, including the higher charge storage capacity of CPs [30]. One study developed a graphene/PEDOT hybrid anode microbial fuel cell (MFC) utilizing *Escherichia coli*, which exhibited improved bacterial colonization and significantly enhanced power generation compared to traditional carbon paper, graphene-modified carbon paper, and PEDOT-modified carbon paper anodes. This research highlights the potential of graphene/PEDOT hybrids as promising materials for MFC anodes [31]. In addition, it was reported that PEDOT/electrochemically reduced graphene oxide/nickel nanoparticle electrodes in the presence of *Escherichia coli* obtained promising results for an *Escherichia coli* MFC with an absolute power and maximum power density of 3.9 ± 0.3 mW and 0.32 mW cm^−2^, respectively. These results highlight the potential of graphene-based composites to improve the performance and power generation capability of MFCs [17].

This study focuses on the characterization and modification of a steel electrode coated with PEDOT and graphene derivatives (GO and rGO) and the investigation of its potential for power generation in a glucose-fed *Methanococcus deltae* cell.

## 2. Materials and Methods

Electrochemical procedures were conducted using a VOLTALAB potentiostat–galvanostat in conjunction with the VOLTAMASTER 4 electrochemical research software. The investigation into sweep speed was conducted utilizing a Gamry INTERFACE 1010E potentiostat–galvanostat, accompanied by the Gamry Instruments Framework electrochemical research software. These setups were chosen for their computer-controlled operation, allowing precise control and the accurate recording of current–potential (I-E) curves and time–current curves (I-t).

### 2.1. Obtaining Modified SS/PEDOT, SS/PEDOT/GO, and SS/PEDOT/rGO Electrodes

All electrochemical investigations were performed under ambient room temperature conditions in an anchored electrochemical cell. In this setup, a saturated calomel electrode (SCE) was used as the reference electrode to maintain a constant reference potential throughout the experiments. In addition, a spiral platinum wire was used as an auxiliary electrode to facilitate the electrochemical reactions.

#### 2.1.1. Synthesis of Graphene Oxide (GO) and Reduced Graphene Oxide (rGO)

The synthesis was carried out using commercial graphite flakes from Sigma-Aldrich^®^ (Munich, Germany) as the initial reagent and a modified Hummer method [32]. A 0.5 g sample of graphite was placed in an ice bath (0–5 °C) and treated with 25 mL of concentrated H_2_SO_4_ (98%) and 0.5 g of KNO_3_ under magnetic stirring. After 10 min, 3.0 g of KMnO_4_ was slowly added to the reaction mixture. The vessel was then transferred to a water bath at 35 °C, and the solution was stirred for 1 h, forming a dark paste. Next, 100 mL of deionized water was added to this solution, and the mixture was stirred at 90 °C for 30 min while 2–4 mL of 30% H_2_O_2_ was added dropwise. The resultant dark brown mixture was allowed to stand for 3 h to facilitate sedimentation. The sediment was then separated by centrifugation, washed with distilled water, and dispersed through gentle ultrasonication for 1 h. This process was repeated several times until a neutral pH was achieved.

The reduction of GO was performed by dissolving 5.0 g of L-ascorbic acid in 50 mL of distilled water. This solution was gradually added to the dispersion of GO and subjected to ultrasonic treatment at 64 °C for 2 h. The reduced product was then washed with distilled water and separated by centrifugation. All reagents used in this study were purchased from Sigma-Aldrich (Munich, Germany).

#### 2.1.2. Preparation of Stainless-Steel AISI 316 (SS) Electrodes as Support

A surface of stainless-steel AISI 316 (SS) was used as the substrate (geometric work area delimited to 1.0 cm^2^); this was conditioned by polishing the surface area with sandpaper of different sizes to remove adherent impurities and homogenize the surface until it had a mirror image. Subsequently, cleaning was carried out with distilled water, high-purity water, and acetonitrile to remove the residues produced by polishing. Cyclic voltammetry (CV) was carried out in acetonitrile (CH_3_CN)-grade HPLC and lithium perchlorate (LiClO_4_). After this process, the SS support was ready for the polymerization process.

#### 2.1.3. Electrosynthesis of SS/PEDOT, SS/PEDOT/GO, and SS/PEDOT/rGO Electrodes

**Scheme 1:**
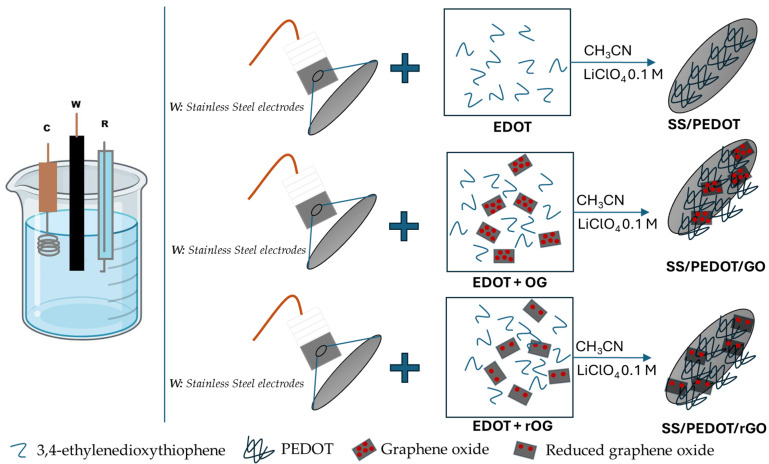
Electrosynthesis of SS/PEDOT, SS/PEDOT/GO, and SS/PEDOT/rGO electrodes.

The electrosynthesis of PEDOT was performed using a potentiostat–galvanostat in CV mode within a three-electrode electrochemical cell. This setup included a saturated calomel electrode as the reference, a coiled platinum wire as the auxiliary (counter) electrode, and an SS sheet as the working electrode (Scheme 1). The electrodes were immersed in an electrolyte solution (10 mL) containing 0.01 M of the monomer 3,4-ethylenedioxythiophene (EDOT) in CH_3_CN, with 0.1 M LiClO_4_ as the supporting electrolyte. The LiClO_4_ concentration was set 100 times higher than that of the monomer to minimize the mass transport of EDOT by migration.

For the electropolymerization of EDOT, different potential ranges from −1.0 V to 1.6 V were evaluated with the number of cycles between 3 and 8 and the scan rate from 0.01 V/s to 0.1 V/s to determine the optimal conditions for the preparation of the modified electrodes. This methodology was extended to synthesize SS/PEDOT/GO- and SS/PEDOT/rGO-modified electrodes by adding 2.0 mg/mL of GO or rGO to the electrolyte solution.

The process was then repeated under the optimal conditions identified for each modified electrode (SS/PEDOT, SS/PEDOT/GO, and SS/PEDOT/rGO) using potential windows of −1.0 V to 1.3 V with 6 cycles, −1.0 V to 1.5 V with 8 cycles, and −1.0 V to 1.6 V with 8 cycles, respectively, and a constant scan rate of 0.10 V/s for all. At this stage, the potential pulse (PP) technique was used to precisely control the nucleation and growth of the polymer to achieve more homogeneous and reproducible surfaces. The perturbation time was studied, starting from an open circuit potential (OCP) of 10 s, followed by 240 s and 1.25 V. After preparation, the electrodes were washed three times with acetonitrile to remove excess monomer and other impurities, and then stored at room temperature for further characterization and application in microbial fuel cells (MFCs).

### 2.2. Characterization of SS/PEDOT, SS/PEDOT/GO, and SS/PEDOT/rGO Electrodes

#### 2.2.1. Electrochemical Characterization

The different surfaces were obtained through the p- and n-type doping/undoping process of the PEDOT polymer in a LiClO_4_ solution [0.1 M] in CH_3_CN using the cyclic voltammetry technique at a scanning speed of 0.01 V/s and 5 cycles. Additionally, the evaluation of this process was carried out using two different electrolytics, which consisted of high-purity water with LiClO_4_ [0.1 M] and with KCl [0.1 M] as supporting electrolytes.

The values for the n-doping/undoping and p-doping/undoping charges shown in Table 1 were calculated from CV data using OriginLab software version 2024. The charge was determined by integrating the current (mA) with respect to time (s) according to the equation Q = ∫I dt, where I represents the current and Q denotes the total charge transferred over time.

#### 2.2.2. Morphological Characterization

The characterization was carried out by means of Raman spectroscopy, using WITec alpha300 RA equipment in single-spectrum mode at various points on the sample surface. Field-emission scanning electron microscopy (FE-SEM) using a Tescan LYRA3 microscope and atomic force microscopy (AFM) using Bruker Innova equipment were also used.

#### 2.2.3. Microbial Fuel Cell Configuration and Operation

*Methanococcus deltae* (ATCC) #35294 cultures were obtained from wastewater from the city of Monteria, Córdoba, Colombia, and isolated in the Biomedical and Molecular Biology Laboratory of Universidad del Sinú (Córdoba, Colombia). To prepare the *Methanococcus deltae* (ATCC) #35294 solution, 50 µL of the microorganism suspension was mixed with 100 µL of glucose [5.55 mM] and 1850 µL of phosphate-buffered saline (PBS = pH 7.4). The cathode chamber was filled with 2000 μL of phosphate-buffered saline.

The MFC was designed and built on an Ender 5 plus 3D printer using polylactic acid (PLA) as the material. This consisted of a rectangle of two compartments separated by a cationic membrane (CMI-7000 Cation Exchange Membranes, Membranes International Inc). The MFC was pretreated with sequential washes with high-purity water and ethanol, respectively. The anode was constructed with the SS/PEDOT, SS/PEDOT/GO, and SS/PEDOT/rGO electrodes, obtained as described in the previous sections. The cathode was constructed with a graphite rod (3.5 cm long, 0.5 cm wide, and 0.5 cm in diameter). The anode chamber was sealed to ensure an anaerobic microenvironment.

To calculate the power, an external resistance of 20 Ω was used, recording a stable potential response. The following equations were used to determine the operation of the constructed MFC.

(1) $$I=\frac{V}{R_{ext}}$$

(2) P=V·I

(3) $$R_{int}=\left(\frac{OCP}{I}\right)-R_{ext}$$

where *I* is the current, *V* is the cell voltage, *R_ext_* is the external resistance, *P* is the power, *R_int_* is the internal resistance, and *OCP* is open circuit potential [33].

## 3. Results

### 3.1. Analysis of Modified SS/PEDOT, SS/PEDOT/GO, and SS/PEDOT/rGO Electrodes

**Figure 1:**
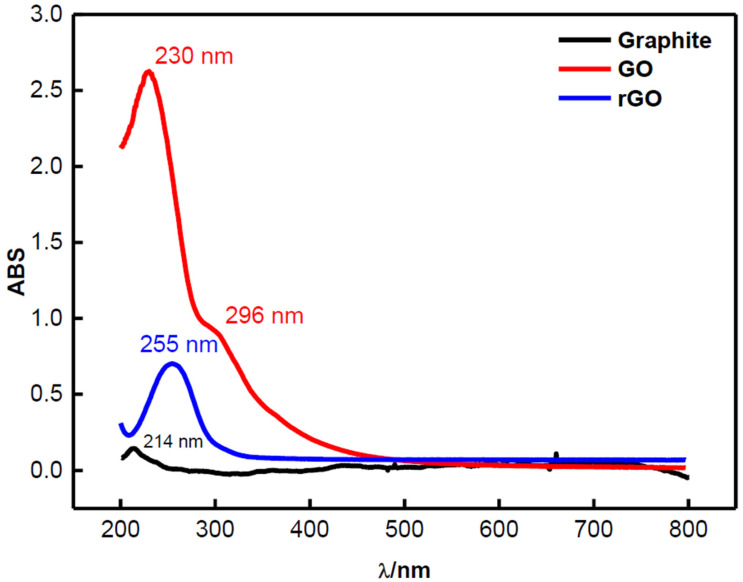
UV–VIS absorption spectrum of graphite, graphene oxide (GO), and reduced graphene oxide (rGO).

After synthesizing graphene oxide (GO) from commercial graphite using the modified Hummer method [32], we proceeded with its characterization using UV–Vis spectroscopy (Figure 1). The spectrum reveals a maximum absorption at approximately 230 nm, which may be due to the π-π * transition of the CC bonds, and a small band at approximately 296 nm, which can be attributed to the n-π * transition of the bonds C=O [34]. Subsequently, GO reduction was performed using an aqueous solution of ascorbic acid and ultrasonication. In the rGO spectrum (Figure 1), a band is detected around 255 nm, while the band at 296 nm is absent, indicating the restoration of double-bond conjugation in rGO sheets and the simultaneous removal of oxygen functionalities [35].

**Figure 2:**
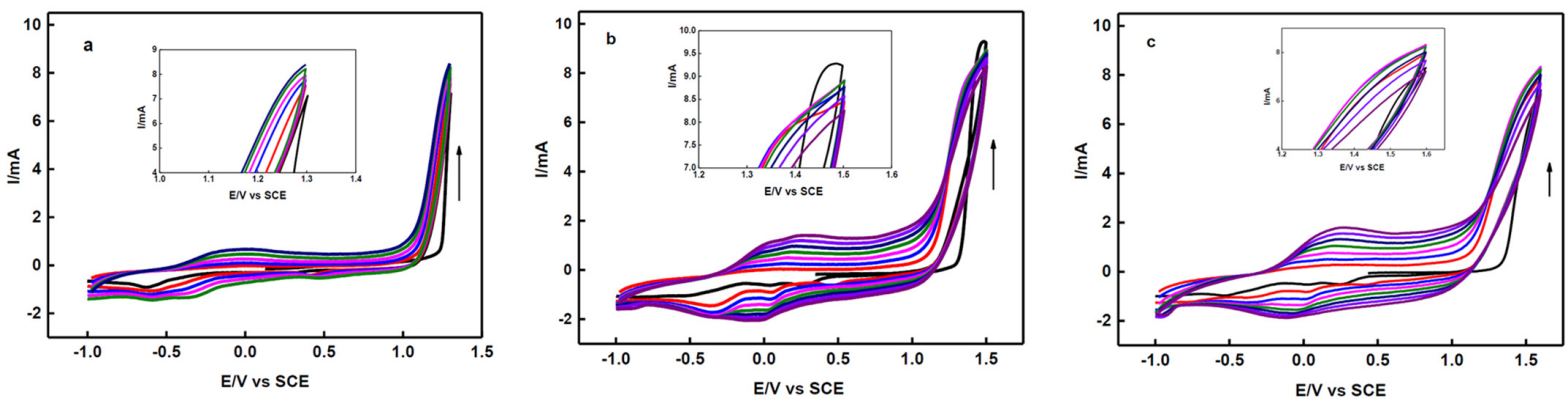
Cyclic voltammogram recorded while obtaining of the electrodes of (**a**) SS/PEDOT n:6, (**b**) SS/PEDOT/GO, and (**c**) SS/PEDOT/rGO n:8 from EDOT 0.01 M using CH_3_CN and LiClO_4_ 0.1 M. Scan rate: 0.10 V/s.

Figure 2 shows the obtained cyclic voltammograms of the electrochemical deposition on the respective electrodes. For each electrode, we optimized the potential window, the scan rate (0.10 V/s), and the number of cycles (n). In all cases, an increase in current is observed as the number of voltammetric cycles increases, as shown in the insets of Figure 2. This behavior is characteristic of the formation of a conductive material on the electronic substrate [36]. A dark deposit consistent with PEDOT polymerization is observed on the SS surface, the thickness of which progressively increases with the number of cycles.

**Figure 3:**
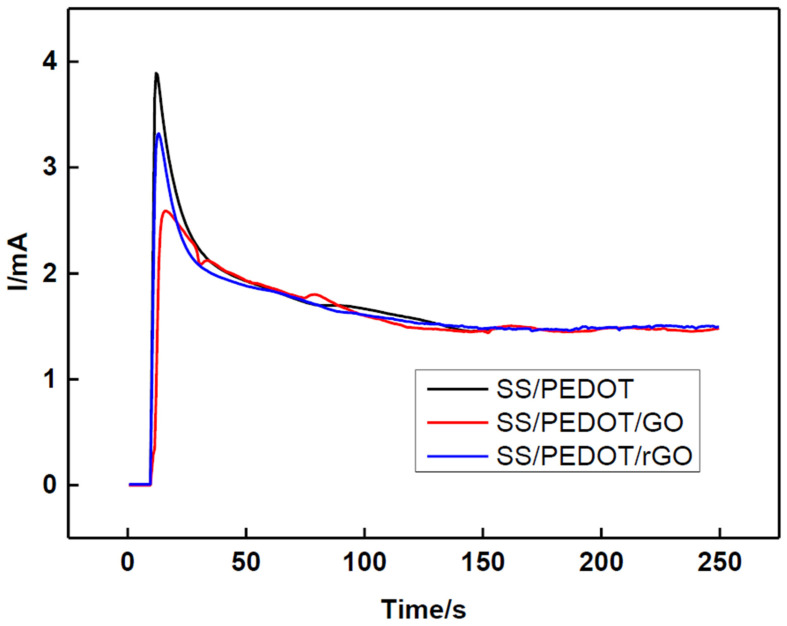
j/t transients obtained during the electropolymerization of EDOT 0.01 M/GO (rGO) at SS with CH_3_CN + 0.1 M LiClO_4_. Polymerization potential, 1.25 V.

After determining the optimal electrosynthesis conditions, electropolymerization was performed using the PP technique to control polymer nucleation and growth. This approach enables the formation of more homogeneous and reproducible modified surfaces, as CV tends to produce irregular surface morphology films. The parameters of applied potential and perturbation time were evaluated, and 1.25 V for 240 s was found to be optimal. Shorter times, such as 60 s, resulted in a thin and transparent film, indicating that the nucleation and growth step of the polymer is just taking place (Figure 3).

In the electrodeposition process, EDOT was oxidized by electrical stimulation at a fixed potential (see Figure 3). This initiates polymerization by generating radical cations, which subsequently facilitate the deposition of the polymeric material on the electrode surface. To maintain electrostatic equilibrium, negatively charged molecules from the polymerization solution are incorporated as dopants to counteract the positive charges along the polymer backbone. The GO (or rGO) molecules have numerous negatively charged carboxylic acid groups on their periphery, which may allow them to be incorporated into the PEDOT film.

### 3.2. Electrochemical Characterization of the SS/PEDOT, SS/PEDOT/GO, and SS/PEDOT/rGO Electrodes

**Figure 4:**
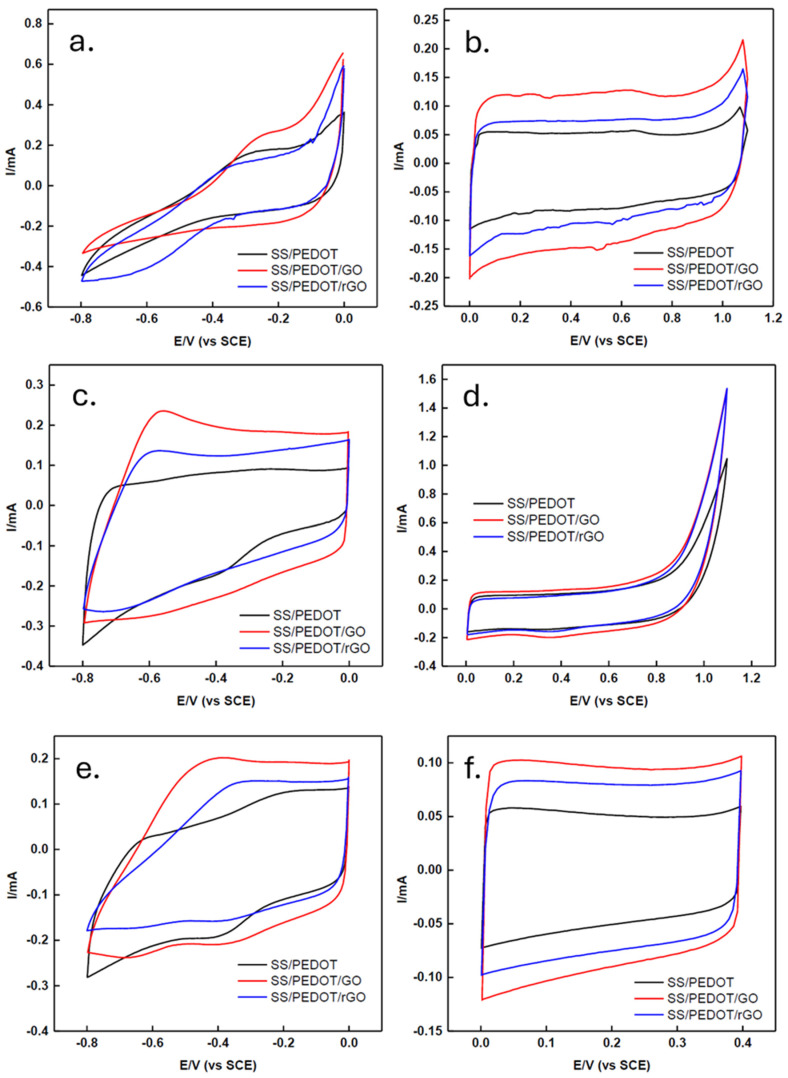
Cyclic voltagrams of the n-doping/undoping and p-doping/undoping processes, respectively. (**a**,**b**): 0.1 M LiClO_4_ in CH_3_CN; (**c**,**d**): 0.1 M LiClO_4_ in H_2_O; (**e**,**f**): 0.1 M KCl in H_2_O. Scan rate: 0.01 V/s, cycle n.

Electrochemical characterization of the SS/PEDOT, SS/PEDOT/GO, and SS/PEDOT/rGO electrodes was performed to evaluate the electrochemical properties and stability of the modified surfaces. This evaluation included p-type and n-type doping/undoping processes using cyclic voltammetry in solutions without the EDOT monomer. The characterization was first performed in an electrolytic medium consisting of CH_3_CN and LiClO_4_, followed by aqueous electrolytes with LiClO_4_ and KCl as supporting electrolytes (Figure 4).

Comparing the voltammetric results of the bare SS (Supplementary Information, Figure S1), SS/PEDOT, SS/PEDOT/GO, and SS/PEDOT/rGO electrodes, it was observed that the SS/PEDOT/GO and SS/PEDOT/rGO electrodes showed significantly higher current response. This improvement is due to the increase in electroactivity provided by the GO and rGO composites integrated with PEDOT, which is consistent with that reported by other authors [37]. In addition, it was verified that the polymer adhered to the surface of the stainless-steel (SS) electrode, as there was no significant decrease in current after five cycles. This indicates that the prepared PEDOT/GO and PEDOT/rGO films have good redox activity and stability. However, this effect is more pronounced for the SS/PEDOT/GO electrode. GO, as an anionic dopant, could react with the ions in the solution. Additionally, GO demonstrates excellent dispersibility due to its highly oxidized structure, which includes various oxygen-containing functional groups (such as alkoxy, epoxy, carbonyl, and carboxyl groups), making this composite more stable in aqueous media.

The electrochemical response of the electrodes was then evaluated using water and acetonitrile as solvents. It was observed that changing the solvent from CH_3_CN to H_2_O increased the redox properties of the material for both p- and n-type doping/undoping (Figure 4 and Table 1), indicating that there is no degradation of the electrode and that these can be used in aqueous media where microorganisms are generally present. However, the change of the supporting electrolyte from LiClO_4_ to KCl resulted in a decrease in the charge obtained for all the electrodes studied, which is attributed to the possible degradation or damage of the composite. The p-doping/undoping process showed a higher charge than the n-doping/undoping process in the studied electrodes, demonstrating that the polymer matrix is easier to oxidize than to reduce. Furthermore, it was established that the SS/PEDOT/GO electrode exhibited the highest charge generation in the studied solutions, with a value of 0.366 mC observed for LiClO_4_/H_2_O.

### 3.3. Morphological Characterization and Chemical Analyses of the SS/PEDOT, SS/PEDOT/GO, and SS/PEDOT/rGO Electrodes

**Figure 5:**
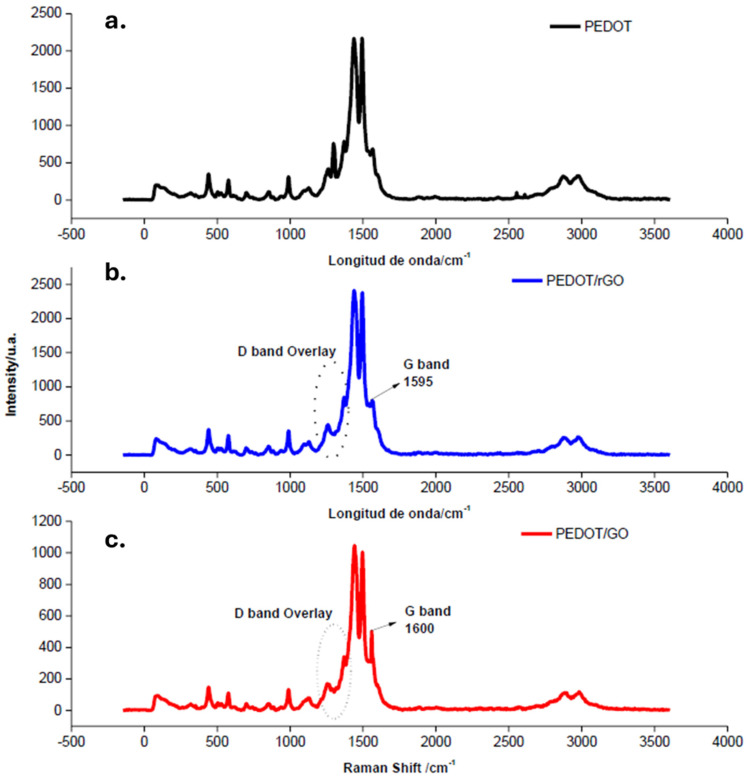
Raman spectrum of electrode surfaces: (**a**) SS/PEDOT, (**b**) SS/PEDOT/rGO, and (**c**) SS/PEDOT/GO.

Morphological characterization and chemical analyses were performed using Raman spectroscopy, FE-SEM, and AFM techniques. In all Raman spectra (Figure 5) of the SS/PEDOT, SS/PEDOT/GO, and SS/PEDOT/rGO electrode surfaces, two bands were observed in the range of 1400–1500 cm^−1^ , related to the symmetric stretching of the Cα=Cβ bond within the thiophene ring. The bands at 438 and 574 cm^−1^ are attributed to the deformation of the CO–C bond. In addition, the vibrational modes observed at 855 and 985 cm^−1^ correspond to the O–C–C deformation and the oxyethylene ring deformation, respectively. These observations provide valuable insights into the dynamic properties of the material, thereby enhancing our understanding of its behavior.

The D-band of GO and rGO is not observed because it overlaps with the PEDOT signals, which show intense signals in the same region. However, in GO, the G-band is blue-shifted up to 1600 cm^−1^ ; this vibration band is due to in-plane stretching of the carbon atom pairs and is observed in all carbon structures containing sp^2^ bonds. In rGO, this band is less intense and red-shifted up to 1595 cm^−1^ , which can be attributed to the reduction of GO. These observations indicate that the graphene derivatives were integrated into the PEDOT matrix (Figure 5b,c).

**Figure 6:**
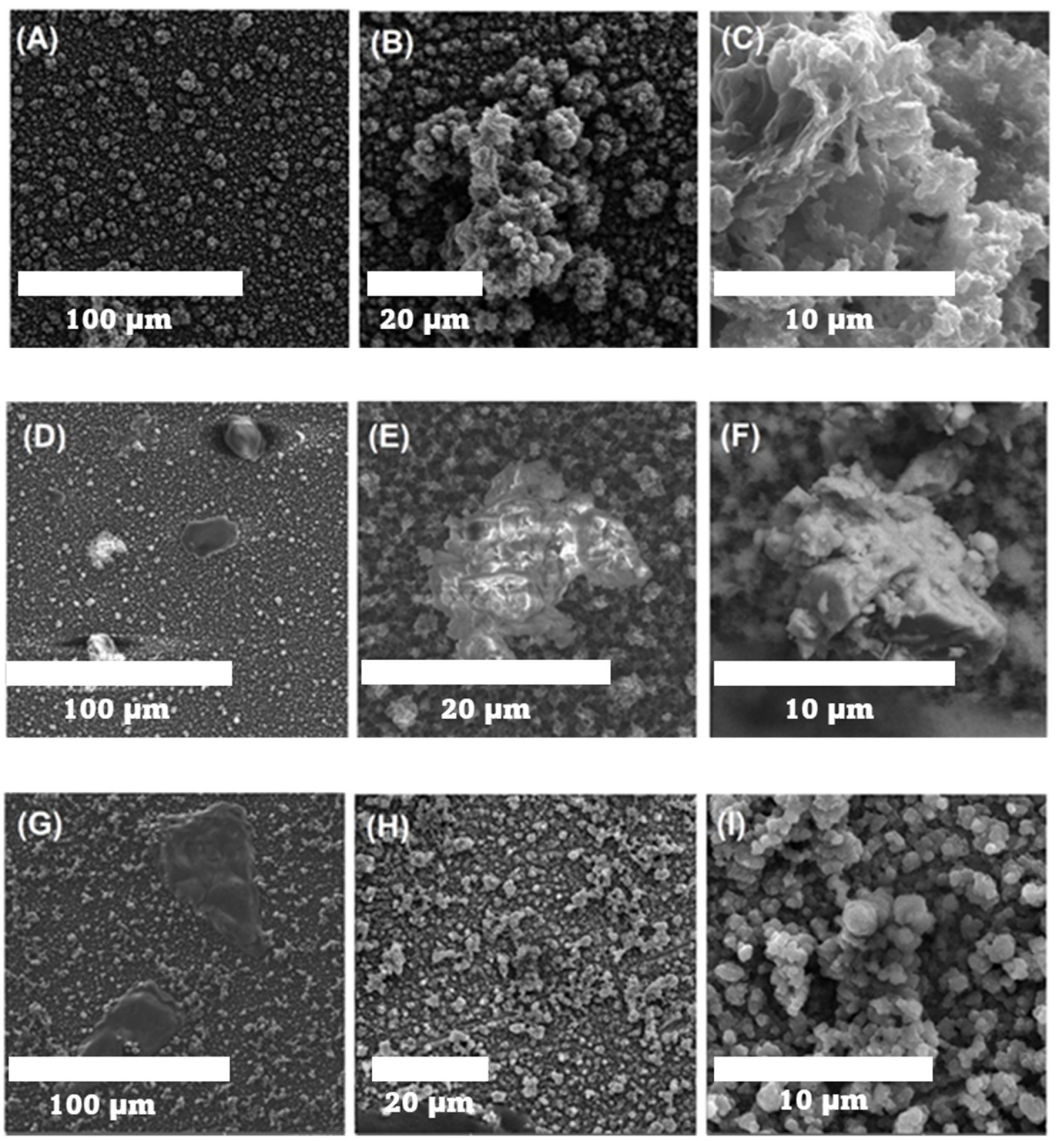
FE-SEM images of films of SS/PEDOT (**A**–**C**), SS/PEDOT/rOG (**D**–**F**), and SS/PEDOT/OG (**G**–**I**) at different scales: 100 μm (**A**,**D**,**G**), 20 μm (**B**,**E**,**H**), and 10 μm (**C**,**F**,**I**).

Field-emission scanning electron microscopy was used to obtain a detailed view of the microstructure of the different SS/PEDOT, SS/PEDOT/rGO, and SS/PEDOT/GO electrodes (Figure 6). The SS/PEDOT electrode surface showed an interconnected network of granular fractal-like structures (Figure 6A–C), and its morphology suggests a high surface area and potential for efficient charge transport. However, when rGO is incorporated into PEDOT (Figure 6D–F), sheets are observed surrounding the PEDOT granules, indicating a high degree of coverage and contact with these granules (Figure 6F). This suggests a significant increase in the specific surface area of the electrode film compared to the PEDOT-only electrode, thereby improving its electrochemical performance. The surface morphology of the SS/PEDOT/GO electrode (Figure 6G–I) is more homogeneous; this difference can be attributed to the ionic interactions between the monomer and the support electrolyte that occur during the electropolymerization process, as well as to the interaction between EDOT and the functional groups in GO, as reported by other authors [38,39]. The functional groups of GO can favor the obtaining of a confined polymeric deposit with small size and uniform distribution, as observed in Figure 6H,I.

**Figure 7:**
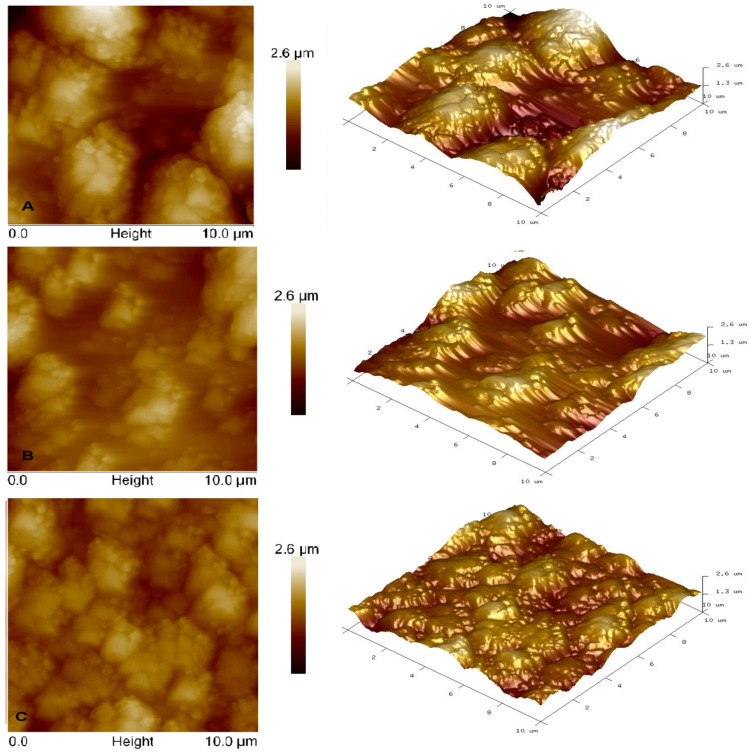
AFM images of films of (**A**) SS/PEDOT, (**B**) SS/PEDOT/rGO, and (**C**) SS/PEDOT/GO.

The topography of the SS/PEDOT, SS/PEDOT/GO, and SS/PEDOT/rGO thin films was analyzed by atomic force microscopy, as shown in Figure 7. The PEDOT surface shows an irregular structure with pronounced valleys (Figure 7A), confirming the presence of granules observed in the FE-SEM images (Figure 6). The SS/PEDOT/rGO surface (Figure 7B) also shows an irregular topography characterized by elevated regions and a relatively flatter surface compared to PEDOT. In contrast, the SS/PEDOT/GO surface (Figure 7C) appears rough with an evenly distributed texture, indicating that the modification of the SS electrode with this composite increases the electroactive area. These AFM images confirm that the incorporation of rGO and GO into the PEDOT electrodes improves both their surface area and electroactivity. A larger electroactive surface area suggests a higher probability of electron capture, which could lead to increased energy output when this modified electrode is used in an MFC.

### 3.4. Electrochemical Description of the SS/PEDOT, SS/PEDOT/GO, and SS/PEDOT/rGO Electrodes in the MFC

**Figure 8:**
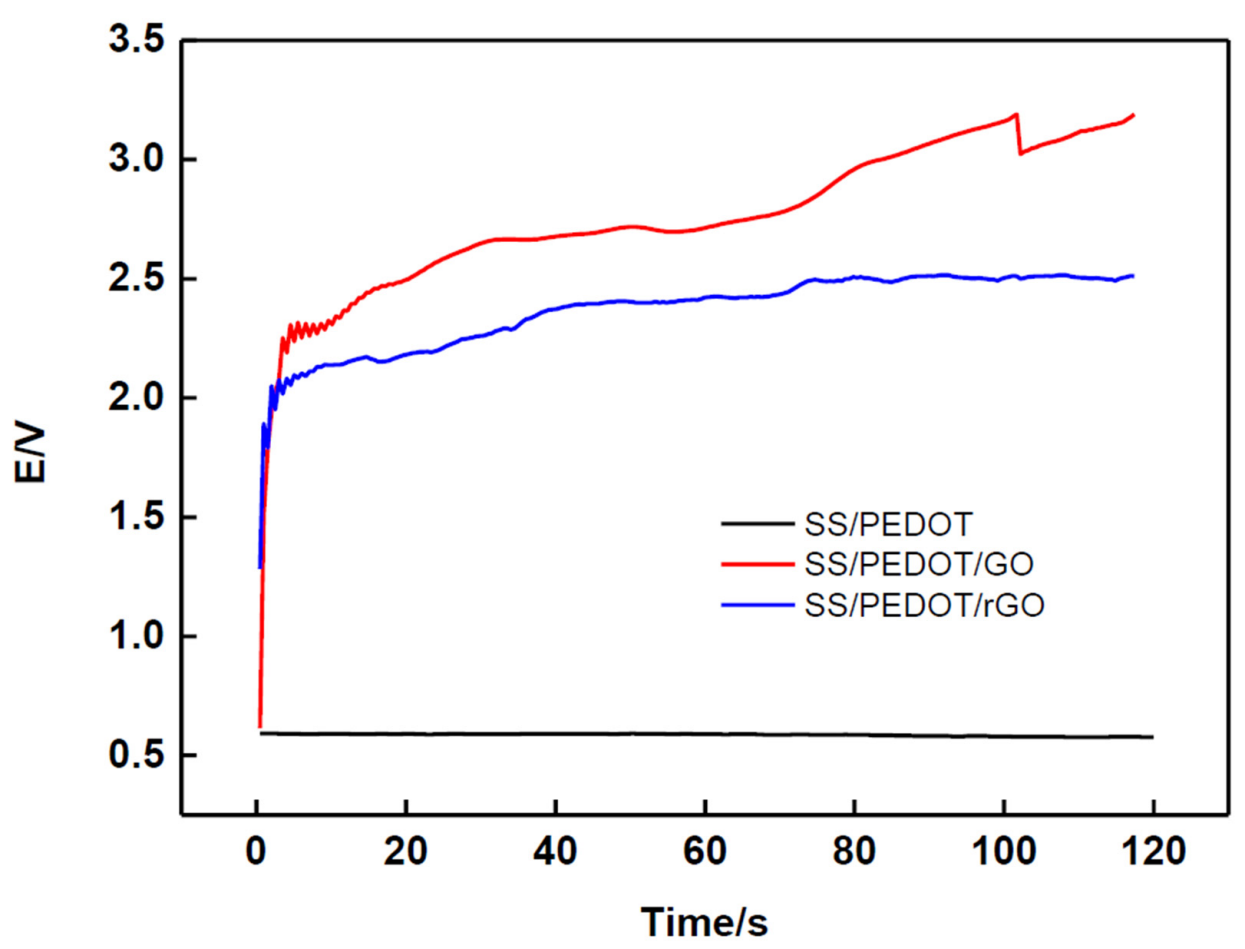
Open circuit potential of the MFC with different compounds as the anode.

The microbial fuel cell is composed of the microorganism *Methanococcus deltae*, utilizing SS/PEDOT, SS/PEDOT/GO, and SS/PEDOT/rGO electrodes as the anode, while a graphite rod serves as the cathode. To evaluate the performance of the MFC, OCP results were compared using SS/PEDOT, SS/PEDOT/GO, and SS/PEDOT/rGO electrodes. The OCP measurements correspond to the potential developed in the absence of current, generated in this case by the activity of *Methanococcus deltae* on the electrode. Figure 8 shows how the MFC potential increased rapidly, reaching values of 0.593, 2.098, and 2.250 V for the SS/PEDOT, SS/PEDOT/rGO, and SS/PEDOT/GO electrodes, respectively. After 120 s, the potential continued to increase to peak values of 2.516 and 3.190 V for the SS/PEDOT/rGO and SS/PEDOT/GO electrodes, respectively, while the potential of the SS/PEDOT electrode remained constant. Although MFCs typically require more time to stabilize the OCV due to biological processes, this study focused on quickly assessing the initial performance of the cells under the specified conditions. The 120 *s* time was sufficient to capture the immediate response of the system and provide valuable insight into the viability and activity of *Methanococcus deltae*. However, it is important to note that these short-term measurements are not necessarily indicative of long-term stability. These OCP values are significantly high, demonstrating the potential of the synergy between the microorganism *Methanococcus deltae* and SS/PEDOT/GO and SS/PEDOT/rGO electrodes as a promising solution for clean energy generation. This increase in performance suggests that the modification of the electrodes with graphene oxide and reduced graphene oxide not only improves the electroactive surface, but also facilitates enhanced interaction between the microorganism and the electrode surface, thus optimizing electron transfer. These results underscore the value of exploring advanced materials in the development of microbial fuel cells, which offer a sustainable and efficient alternative form of renewable energy production.

Table 2 summarizes the electrical characteristics of each surface evaluated. It is observed that the maximum power density obtained was 34.394, 1014.420, and 632.019 mW/cm^2^ for the SS/PEDOT, SS/PEDOT/GO, and SS/PEDOT/rGO electrodes, respectively. These results indicate that the incorporation of GO and rGO on the anodic surface improves the power generation of the system. The most effective electrode was the one composed of SS/PEDOT/GO, achieving the highest power density (1014.420 mW/cm^2^) and outperforming the SS/PEDOT and SS/PEDOT/rGO electrodes. This high power density can be attributed to several synergistic factors occurring at the anode. First, the π-π interaction between the PEDOT chain and the GO films favors adsorption and coupling. This interaction stabilizes the anodic structure and optimizes the charge distribution at the electrode–electrolyte interface, improving the efficiency of the electron transfer process. In addition, GO acts as an effective electron bridge between the microorganism and the electrode surface, allowing the electron transfer rate to increase and reducing the internal resistance of the system [40].

On the other hand, although the SS/PEDOT/rGO electrode also showed a significant improvement in power density compared to SS/PEDOT, its performance was lower than that of the SS/PEDOT/GO electrode. This suggests that although the reduction of GO to rGO improves some electrical properties [41,42], it may decrease the ability of the material to interact with microorganisms, reducing the active sites available for adsorption.

## 4. Conclusions

The successful fabrication and characterization of PEDOT/GO and PEDOT/rGO composites on SS surfaces was achieved using electrochemical techniques (CV and PP), spectroscopic methods (UV–Vis and Raman), and FE-SEM and AFM for morphological and topographical analysis, respectively. The composites showed effective doping/undoping behavior in both p- and n-type processes with different solvents (CH_3_CN, H_2_O) and supporting electrolytes (LiClO_4_, KCl). Among the electrodes tested, SS/PEDOT/GO showed superior electrochemical performance. The electrochemical evaluation of the MFC-modified electrodes revealed potentials of 2.516 V and 3.190 V for the SS/PEDOT/rGO and SS/PEDOT/GO electrodes, respectively, highlighting the synergistic interaction between the microorganism Methanococcus deltae and these electrode materials. In particular, the SS/PEDOT/GO electrode achieved the highest power density (1014.420 mW/cm^2^), demonstrating its potential as a highly effective component in MFC applications. These findings open the way for future research to further explore material combinations and surface modifications to improve MFC performance and durability.

## Figures and Tables

**Table 1:** n-Doping/undoping and p-doping/undoping charges for the electro-obtained surfaces with SS/PEDOT, SS/PEDOT/GO, and SS/PEDOT/rGO.

**n-Doping/Undoping**			
**Electrode/electrolyte solution**	**LiClO_4_/CH_3_CN (mC)**	**LiClO_4_/H_2_O (mC)**	**KCl/H_2_O (mC)**
**PEDOT**	0.161	0.180	0.179
**PEDOT/GO**	0.210	0.289	0.246
**PEDOT/rGO**	0.181	0.214	0.167
**p-Doping/Undoping**			
**Electrode/electrolyte solution**	**LiClO_4_/CH_3_CN (mC)**	**LiClO_4_/H_2_O (mC)**	**KCl/H_2_O (mC)**
**PEDOT**	0.139	0.277	0.040
**PEDOT/GO**	0.276	0.366	0.073
**PEDOT/rGO**	0.189	0.286	0.060

**Table 2:** Data obtained by means of electrochemical characterization of the MFC with the different electrodes.

Electrode	Potential	Current	Power Density	Internal Resistance
	(V)	(A)	(mW/cm^2^)	(Ω)
PEDOT	0.593	0.029	34.394	0.448
PEDOT/GO	3.190	0.159	1014.420	0.063
PEDOT/rGO	2.516	0.126	632.019	−0.032

## Data Availability

Data are contained within the article and Supplementary Materials, further request please contact the corresponding author.

## Supplementary Materials

The following supporting information can be downloaded at: https://www.mdpi.com/article/10.3390/polym16223134/s1, Figure S1: Cyclic voltagrams of the a. n-doping/undoping process and b. p-doping/undoping process of SS/PEDOT in 0.1 M LiClO4 (CH3CN). Scan rate: 0.01 V/s; n = 5 cycles; the last cycle is labelled.
