# Supplementary material for: Design and Optimization of PEDOT/Graphene Oxide and PEDOT/Reduced Graphene Oxide Electrodes to Improve the Performance of Microbial Fuel Cells, Accompanied by Comprehensive Electrochemical Analysis
Source: Polymers (Basel). 2024 Nov 10;16(22):3134. doi: 10.3390/polym16223134 (PMC11598706; doi:10.3390/polym16223134)
Supplement: Supplementary file 1 [file polymers-16-03134-s001.zip › polymers-3294200-supplementary.pdf]

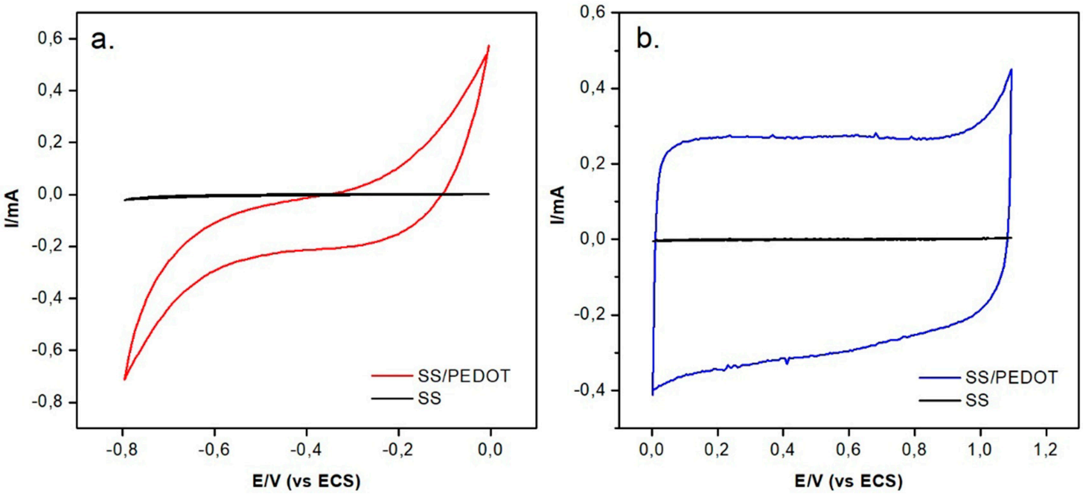

Figure S1. Cyclic voltagrams of the a. n-doping/undoping process and b. p-doping/undoping process of SS/PEDOT in 0.1 M LiClO4 (CH3CN). Scan rate: 0.01 V/s; n = 5 cycles; the last cycle is labelled.
